# Supplementary material for: Machine-supported decision-making to improve agricultural training participation and gender inclusivity
Source: PLoS One. 2023 May 5;18(5):e0281428. doi: 10.1371/journal.pone.0281428 (PMC10162538; doi:10.1371/journal.pone.0281428)
Supplement: S1 File — (DOCX) [file pone.0281428.s001.docx]

**Supplemental Information**

**Part A: Performance comparison of three model shrinkage approaches**

The original analysis in the paper randomly divides the dataset into a 80/20 split, where 80% was considered as the training dataset and the 20% holdout data was considered as the testing dataset. The procedure in the paper was only perform once. In the following analysis, the same procedure was repeated ten times and for each repetition, the mean squared error was evaluated using the testing dataset for the model constructed using (i) group lasso, (ii) group minimax convex penalty (MCP), and (iii) group smoothly clipped absolute deviations penalty (SCAD). Figure S.1 shows the performance of the three methods, evaluating the mean square error in the testing dataset for each of the ten repetitions.

For this particular dataset, there was not a shrinkage method that provided the lowest mean square error in the testing dataset for all repetitions. However, on average, the MCP method provided slightly lower mean square error, while using the fewest model parameters. In future research, it is recommended to include this step in order to select the model shrinkage procedure that performs the best given the data characteristics.


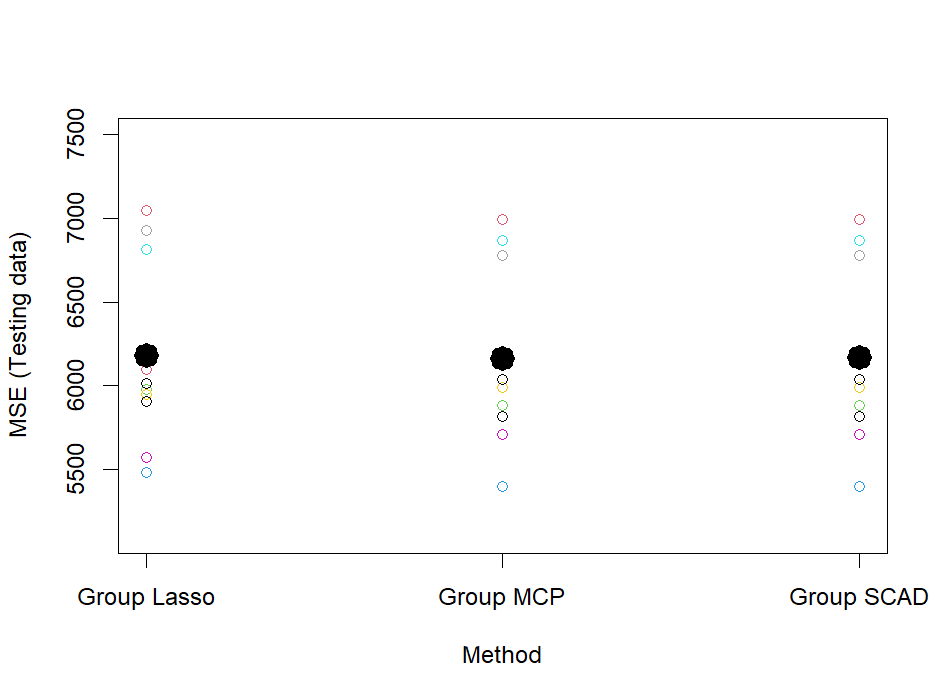


Figure S.1 Mean square error (MSE) in the testing dataset for the three model shrinkage procedures: Group lasso, group minimax convex penalty (MCP), and group smoothly clipped absolute deviations penalty (SCAD).

**Part B: Performance comparison between a simple regression approach with a more advance machine learning approach**

In the previous publication^1^, humans selected the parameters to be incorporated into the regression model, focusing only on main effects and their associations with total attendance and female participation. In the current manuscript, the scope of the regression model was expanded to include main effects and their interactions. We then allowed the machine using model shrinkage to select a smaller subset of parameters that were important to predict total attendance and female participation. Model shrinkage was used to improve the generalizability of the model by avoiding over-fitting of data.

In order to compare the more simple regression approach (i.e., human selected main effects) with the more advanced machine learning approach (i.e., machine selected subset of main effects and interactions), the following procedures were followed:

1. Divide the data in training and testing datasets. The percentage of the full dataset to be used as the training dataset was 90%, 85%, 80%, 75%, 70%, and 65%.
2. Using the training dataset, create models using Poisson regression and negative binomial regression.
3. Using the testing dataset, evaluate the prediction error for each model type (Poisson, negative binomial regression) using the various percentages of data allocated to the training dataset (90%, 85%, 80%, 75%, 70%, and 65%).

Steps 1, 2, and 3 were repeated 1000 times and the average mean square error was used to evaluate the performance of each model type and data allocation percentage for training/testing.

Results show that the machine learning approach generated less error than the previous regression model for predicting male attendance (Table S1) and slightly less error for predicting female attendance (Table S2). We suspect that broadening the model to include interactions was more benefical for the male model than the female model, which did not incorporate any interactions. In the male model, there was an interaction between Bangladesh division and time of day, suggesting that the best time of day for training to increase male turnout varied amongst divisions. For females, the best time of day for training appears to be more consistent across divisions. Given that error was consistently reduced in both the male and female model, regardless of model type and data allocation percentage, the results support the use of the machine learning approach in the current paper.

Table S.1. Simple regression versus machine learning approach comparison of average mean squared error in predicting the number of males attending training in the testing dataset for different data allocation percentages.

| Outcome: Number of males | | |
| --- | --- | --- |
| Percentage of data allocated as training dataset | Model 1: Parameters selected by humans^1^ | Model 2: Parameters selected by a machine using the Minimax Convex penalty (MCP) shrinkage approach |
| Model: Poisson regression | | |
| 90% | 6717.25 | 6456.65 |
| 85% | 6634.43 | 6374.48 |
| 80% | 6698.51 | 6426.83 |
| 75% | 6707.06 | 6435.05 |
| 70% | 6719.38 | 6431.31 |
| 65% | 6740.86 | 6448.77 |
|  |  |  |
| Model: Negative binomial regression | | |
| 90% | 6777.260 | 6487.989 |
| 85% | 6777.260 | 6403.568 |
| 80% | 6748.517 | 6453.801 |
| 75% | 6769.570 | 6466.826 |
| 70% | 6771.374 | 6463.887 |
| 65% | 6794.195 | 6477.750 |

Table S.2. Simple regression (model fitting) versus machine learning approach (model shrinkage and fitting) comparison of average mean squared error in predicting the number of females attending training in the testing dataset for different data allocation percentages.

| Outcome: Number of females | | |
| --- | --- | --- |
| Percentage of data allocated as training dataset | Model 1: Parameters selected by humans^1^ | Model 2: Parameters selected by a machine using the Minimax Convex penalty (MCP) shrinkage approach |
| Model: Poisson regression | | |
| 90% | 419.73 | 416.14 |
| 85% | 422.65 | 419.02 |
| 80% | 425.41 | 421.02 |
| 75% | 424.49 | 419.45 |
| 70% | 423.68 | 418.30 |
| 65% | 424.89 | 418.83 |
|  |  |  |
| Model: Negative binomial regression | | |
| 90% | 457.55 | 432.93 |
| 85% | 463.87 | 437.51 |
| 80% | 466.76 | 439.71 |
| 75% | 467.75 | 438.38 |
| 70% | 469.53 | 437.28 |
| 65% | 473.71 | 439.14 |

**Part C: Performance comparison between regionally unbalanced versus balanced training selection**

Simulations were performed using the machine learning approach to determine the impact of different training selection policies. For these simulations, the machine learning approach used Minimax Convex Penalty shrinkage to select parameters and used 80% of the data for model training and 20% for testing. The two training selection policies included 1) regionally unbalanced training selection in which training is selected from among the top training events irrespective of division, and 2) regionally balanced training selection in which training events are balanced between divisions. The unbalanced training selection policy is expected to increase general training turnout and female participation; however, the balanced training selection policy may be more desirable allowing for more equitable access to agricultural extension training. The objective of these simulations is to gain insight into the performance decrement that would be incurred by balancing training across all divisions.

For unbalanced training selection, the top 24 training events for the total attendance and the top 24 training events for female attendance were selected, irrespective of division. While for balanced training selection, the top training lists for each division were created and used for training selection. Six of the top total training events and 6 of the top female training events were selected for each of the 4 divisions. Prior analysis suggests that midsize lists (e.g., lists of 50) with an equal selection from the top training and top female training events was desirable. This training selection policy produced total and female attendance that exceeded past training benchmarks based on human intuition, while allowing for sufficient training variety to promote future machine learning.

To evaluate the performance of the regionally unbalanced versus balanced training selection policies, the estimated average attendance along with 95% confidence intervals were calculated. As expected, regionally unbalanced training selection yielded a higher average total attendance and female attendance, but not substantially better than balanced training selection (140 vs 136 total attendees per event and 41 vs 37 female attendees per event, see Figure S2). Moreover, the balanced training selection exceeded benchmarks based on prior training selection using human intuition. These results suggest that more equitable access to agricultural training selection does not negatively impact training turnout and gender inclusivity.


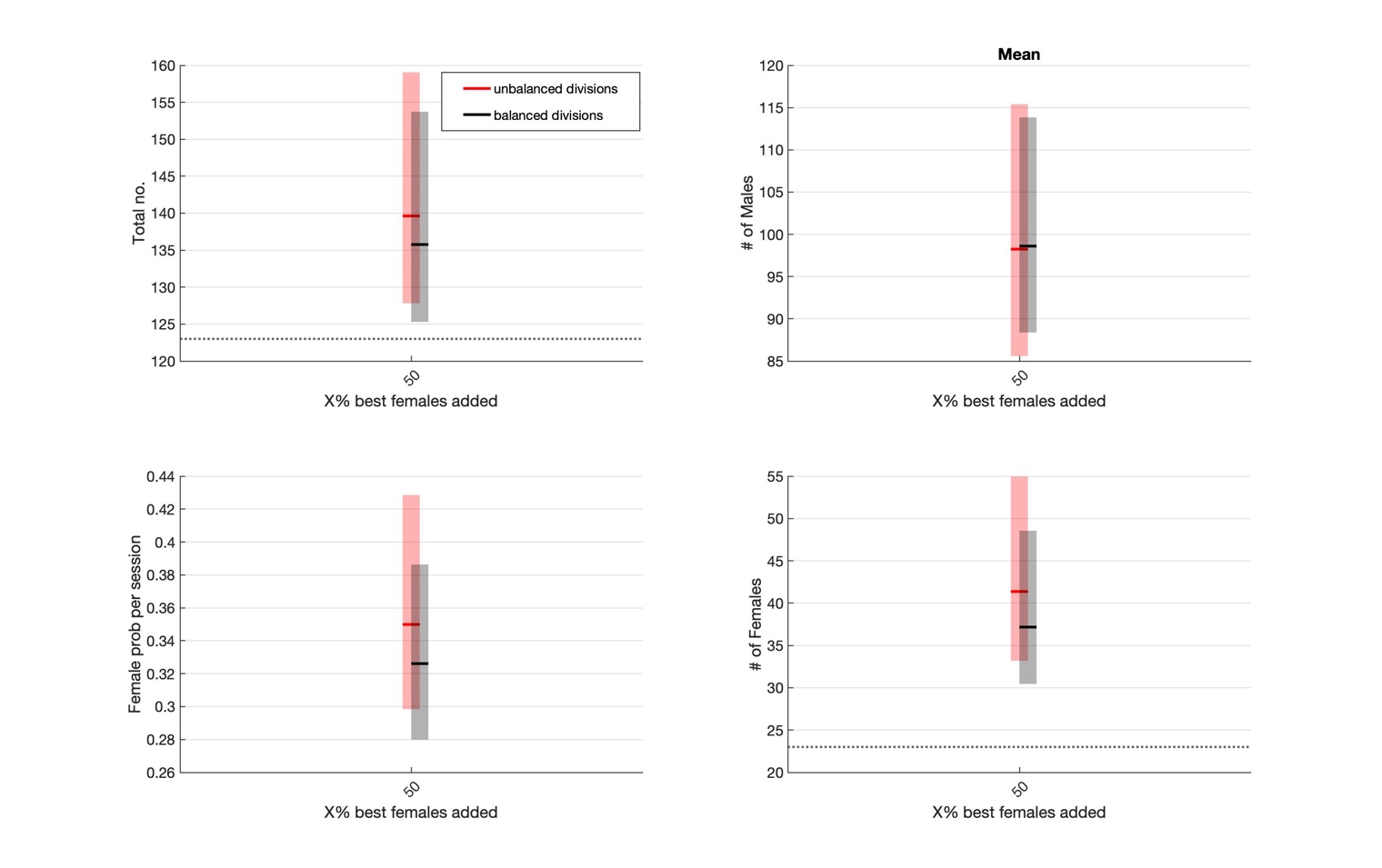
Figure S.2: Unbalanced versus balanced training selection simulations for average total attendance (top-left), average male attendance (top-right), and proportion of female attendance (bottom-left), and average female attendance (bottom-right). Red represents unbalanced training selection (selecting the best training events, irrespective of division); black represents balanced training selection amongst divisions. As a benchmark, the dotted line represents the average total attendance (122 persons per event) and female attendance (23 females per event) when selecting training events using human intuition. The goal is for the lower 95% limits to exceed these averages, thus inidicating that machine supported decision making can help improve training attendance and gender inclusivity.

Reference

1 Medendorp, J. W. *et al.* Large-scale rollout of extension training in Bangladesh: Challenges and opportunities for gender-inclusive participation. *PLoS One* **17**, e0270662, doi:10.1371/journal.pone.0270662 (2022).
