# Supplementary material for: Machine-supported decision-making to improve agricultural training participation and gender inclusivity
Source: PLoS One. 2023 May 5;18(5):e0281428. doi: 10.1371/journal.pone.0281428 (PMC10162538; doi:10.1371/journal.pone.0281428)
Supplement: Attachment — Submitted filename: Reviewer Response pr14 gc2.docx [file pone.0281428.s002.docx]

**PlosOne**

**Point-by-Point Response to Reviewers**

PONE-D-22-16835

Data-driven Modeling to Improve Agricultural Training Participation and Gender Inclusivity

*We thank the editors and reviewers for their thoughtful comments. Significant changes to the paper were made based on these comments (see the point-by-point response below). Our comments are in italics. Parts taken from the paper are in quotation marks (“ ”), and new sections added are underlined.*

**Responses to Editor's Comments:**

1) The paper presentation should be cared more, in particular it should be provided clearer motivations.

2) A reviewer criticizes also the cited body of research, which looks not to be exhaustedly covered, and he suggests to enlarge explicitly the "Related Work" section.

3) You should pay attention to provide more motivation and description on the used methodology, providing a clearer validity about the overall approach.

*We agree that the motivation for the paper was not clear, which then has led to issues with the literature search and methodology. It appears that reviewer #1 was expecting a different type of paper, possibly one that tests specific training hypothesis (e.g., female-led training events increase female turnout). This was not the focus of the current study. A prior publication that was submitted with the current manuscript evaluates different factors on training turnout and the probability of females attending training, and provides a more exhaustive literature review related to such factors, and provides specific policy recommendations. In this prior study, important lessons were learned including that factors affecting general turnout and gender inclusivity are multifactorial, making designing effective agricultural extension programs challenging, especially when multiple program objectives are present (e.g., increase total training turnout while increasing female turnout concurrently). In the introduction of the current paper, we make the case that such decisions are beyond human intuition and instead require more formal mathematical approaches involving data-driven modeling. This was the focus of the current study and was formally stated in the hypothesis. With that said, we agree with the reviewer that the motivation for the current study needs to be more clearly defined in the introduction to correctly set the expectations of readers. We have made the following changes to the introduction to address this point.*

*"While ICTs increase access to agricultural extension services for production (**17**,* *18**), their deployment must take into account gender-based training preferences. Recent analysis of ICT-delivered agricultural training indicates that differences in male and female farmers exists (**19**). This study as well as others have shown that male and female farmers may differ in terms of when and where they prefer training to be conducted and whether training is male- or female-led (**20-24**). As such, choosing times in the day, days of the week, locations and venues, and gender of the trainer that increased male turnout may decrease the probability of females attending training, and vice versa (**19**). Therefore, designing effective agricultural training programs that may include multiple objectives, (e.g., increasing general attendance while maintaining a certain proportion of female participation), may not be trivial."*

*" ... We hypothesize that machine supported decision making that accounts for gender-based training preferences can concurrently increase total and female participation over that of the current approach that relies only on human intuition. If results support this hypothesis, incorporating machine learning as part of ICTs, and other modes of information dissemination, in large-scale agricultural extension programs is warranted. "*

*In terms of the literature, the following section was added to the introduction.*

*"Although there is considerable research into the use of machine learning in agriculture, the applications mainly focus on crop, livestock, water and soil management (**25**); there does not appear to be applications using data-driven modeling to improve agricultural extension participation, including promoting gender inclusivity."*

*In terms of the methodology, appropriate modeling approaches were applied to test the hypothesis that machine supported decision making can lead to greater total participation as well as higher female participation than the current approach that relies only on human intuition. We also demonstrate how model simulations can be used to design sensible gender inclusivity goals and lay the foundation for future implementation by exploring the trade-off between model performance and machine learning.*

4) Explain better and motivate the employed dataset and features, in particular to the data used for the training split.

*The first stage of the analysis was to decide among three model selection techniques (model shrinkage for variable selection). For that purpose, data was divided in two pieces: training (80% of the dataset) and testing (20% of the dataset). The randomly selected training sample was used for the three techniques to present their proposal and the testing dataset was used to compare the performance of the three using mean squared error. It is important to mention that all three model shrinkage techniques use cross validation, which is common to select the optimal shrinkage parameter (31).*

*This has been clarify in the paper.*

"*To develop more parsimonious models to improve precision in model predictions, the number of model inputs were reduced for the two gender specific training models using a shrinkage approach. Three different shrinkage approaches were considered: group Lasso (group Lasso), group Minimax Convex Penalty (group MCP), and group Smoothly Clipped Absolute Deviation (group SCAD). Group selection was chosen since several model inputs were grouped by factors (time of the day, day of the week, month of the year, venue type, and Bangladesh division). All shrinkage approaches used datasets for learning (randomly selected 80% of data) and testing (remaining 20% of data) to train and evaluate the gender specific training models. All three model shrinkage approaches used cross-validation in order to select the optimal shrinkage parameter (**31**)."*

**Journal Requirements:**

1. Please ensure that your manuscript meets PLOS ONE's style requirements

*We have reviewed the style requirements and have made significant changes to the manuscript, including reorganizing the Materials and Methods section to be more concise.*

2. Please include a complete copy of PLOS’ questionnaire on inclusivity in global research in your revised manuscript.

*We completed the PLOS' questionnaire on inclusivity in global research. Only the general section needed to be completed. We used the NIH definition of human research to determine if the current study represents human research, which would then require the section on human research to be completed. Our study is not considered human research given that (1) the study involves only secondary research using data not collected specifically for this study, and (2) data was provided without identifiable information.*

*As indicated in the manuscript, "This study was deemed exempt by Michigan State University Biomedical and Health Institutional Review Board (IRB 00004626)."*

*The following was also added to the Materials and Methods section.*

*"Inclusivity in global research.*

*Additional information regarding the ethical, cultural, and scientific considerations specific to inclusivity in global research is included in the Supporting Information (SX Checklist)."*

3. Thank you for stating the following in the Competing Interests section:

“I have read the journal's policy and the authors of this manuscript have the following competing interests:

N. Peter Reeves is the Founder and President of Sumaq Life LLC.  Ahmed Ramadan is a part-time employee of Sumaq Life LLC.  Sumaq Life LLC applies mathematical modeling approaches to understand complex systems in order to optimize their performance.  It receives funding for these services, including work on the current project. The remaining authors declare no competing interest in the production of this work.”

Please confirm that this does not alter your adherence to all PLOS ONE policies on sharing data and materials, by including the following statement: "This does not alter our adherence to  PLOS ONE policies on sharing data and materials.” (as detailed online in our guide for authors <http://journals.plos.org/plosone/s/competing-interests>).  If there are restrictions on sharing of data and/or materials, please state these. Please note that we cannot proceed with consideration of your article until this information has been declared.

Please include your updated Competing Interests statement in your cover letter; we will change the online submission form on your behalf.

*An updated Competing Interests statement was provided in the cover letter.*

4. Thank you for stating the following in the Acknowledgments Section of your manuscript:

“This work was supported by the Borlaug Higher Education for Agricultural Research and Development Program (BHEARD) and USAID under Grant # AID-BFS-G-11-00002 and #BFS IO-17-00005; and the Bill and Melinda Gates Foundation support for the Cereal Systems Initiative for South Asia (CSISA) under Grant # BMGF INV-009787. Its contents are solely the responsibility of the authors and do not necessarily represent the official views of USAID, BHEARD, CSISA, or the Bill and Melinda Gates Foundation.”

We note that you have provided additional information within the Acknowledgements Section that is not currently declared in your Funding Statement. Please note that funding information should not appear in the Acknowledgments section or other areas of your manuscript. We will only publish funding information present in the Funding Statement section of the online submission form.

Please remove any funding-related text from the manuscript and let us know how you would like to update your Funding Statement. Currently, your Funding Statement reads as follows:

“This publication was made possible in part by internal funds from Michigan State University [JBB & BRP]. The fall armyworm animation and scaled extension activities were supported by funds provided to CIMMYT by the Borlaug Higher Education for Agricultural Research and Development Program (BHEARD; USAID award # AID-BFS-G-11-00002) at Michigan State University [JWM] and the Bill and Melinda Gates Foundation support for the Cereal Systems Initiative for South Asia (CSISA; USAID award #BFS-IO-17-00005 and BMGF isINV-009787)[TJK].

USAID: <https://www.usaid.gov>

BMGF: <https://www.gatesfoundation.org>

Its contents are solely the responsibility of the authors and do not necessarily represent the official views of USAID, BHEARD, CSISA, or the Bill and Melinda Gates Foundation.

No sponsors or funders played any role in the study design, data collection and analysis, decision to publish, or preparation of the manuscript.”

Please include your amended statements within your cover letter; we will change the online submission form on your behalf.

*The acknowledgement section was removed from the manuscript. No changes to the current funding statement will be made.*

5. We note that you have stated that you will provide repository information for your data at acceptance. Should your manuscript be accepted for publication, we will hold it until you provide the relevant accession numbers or DOIs necessary to access your data. If you wish to make changes to your Data Availability statement, please describe these changes in your cover letter and we will update your Data Availability statement to reflect the information you provide.

*No changes to the Data Availability statement will be made. The data will be made available to the public and we will provide the relevant accession number.*

**Responses to Reviewers Comments**

**Reviewer #1:**

1) I am delighted to review the article entitled “Data-driven Modeling to Improve Agricultural Training Participation and Gender Inclusivity”.

*Thank you. We appreciate your constructive feedback and dedication to reviewing the manuscript.*

2) Over all this study have potential implications, but it needs to be improved in several parts, and I have noticed some typo errors throughout the manuscript.

*We apologize for having some grammatical errors. We have reviewed the manuscript for typos prior to resubmission.*

3) The survey methods and data used in this manuscript seems inadequate to be published in this high ranked journal, it still needs to be comprehensively explained with the clearer objectives.

*This study uses preexisting data that was captured, not specifically for research purposes, but instead as part of an extension outreach program. With that said, this real-world data, used to generate data-driven models, provides useful insights into how best to design extension training programs to increase general turnout and gender inclusivity. Further, the data provides a nice starting point for model development, and the model predictions, for the most part, match observed data, suggesting the models are accurate, despite the limitations in the data collection methods. However, we agree with the reviewer that a more rigorous surveying method, while including more variables of interest, should be incorporated into extension training programs in the future.*

*The following was added to the discussion.*

*"To facilitate machine learning, continuous sampling and processing of data of agricultural training programs are recommended. That is (1) host training events, (2) learn (computationally) from those events, (3) enact recommendations for the next set of training events, and repeat to optimize outcomes. In subsequent rounds of data collection, the project could shift from being purely observational to using optimal experimental design (**33**) to focus data collection to help refine models further and to improve their performance. "*

4) The analytical approach needs to be improved and shorten to specific points.

*We are not clear what specifically needs to be improved with the analytical approach. We were intentional in the type of modeling approach used in the paper and used novel methods for shrinking models, which was required to avoid over-fitting of data and to identify those model inputs that have a strong effect on training turnout and female participation. The use of bootstrapping was required to create point estimates and 95% confidence intervals to better understand if certain training events were significantly better than others. The use of simulations were used to (1) help design sensible gender inclusivity policies, and (2) explore the trade-off between model performance and machine learning, which given the early stages of this line of research is warranted.*

*Based on the reviewer's comment, significant changes to the Materials and Methods section were made to shorten it and improve its readability.*

5) I do appreciate that the topic may have potential connections to ethics literature, but unfortunately at present these areas are not well developed.

*Thank you for this comment. As model simulations show, detrimental unintended consequences can occur, such as a significant decrease in overall turnout, if too much focus is placed on increasing female participation in training. Policy makers will have to make difficult decisions regarding how much effort should be placed into increasing female participation, but having machine supported decision making could help policy makers design sensible gender inclusivity goals for large-scale extension training programs.*

*The following was added to the discussion to highlight this important topic raised by the reviewer.*

*"The objective of this study was to determine if machine supported decision making could improve training attendance and gender inclusivity, which would then justify its use in designing large-scale agricultural training programs. The results suggest that, if gender inclusivity goals are sensibly designed, machine supported decision making can concurrently increase total attendance and gender inclusivity. The results, however, also show that overly emphasizing female participation can have negative consequences for total training turnout. For instance, selecting all the training events from the top female list would increase female attendance and the proportion of female attendance to ~ 0.5 but could decrease total attendance below that of the benchmark average of 122 attendees per event (Figure 1). Moreover, many of the top training events for females are predicted to take place in Rajshahi, and therefore, focusing on this one division may be beneficial for overall female attendance, but it would limit gains in female participation to one region, which may not fulfil the goals of the extension program. Machine supported decision making could help address some of these potential ethical dilemmas that policy makers face when creating gender inclusivity goals by allowing them to first simulate "what if" scenarios before applying policies in the real-world."*

6) Regarding contributions, at the end of the paper your discussion appears rather descriptive about what you have done in your study. As such, your paper appears rather limited in terms of addressing or elaborating a specific theoretical/ethics puzzle that would appeal to the audience/readers.

*Based on the reviewer's comment, we reference our prior study that elaborates on male and female preferences for training and that provides some policy recommendations.*

*"Recent analysis of ICT-delivered agricultural training indicates that differences in male and female farmers exists (**19**). This study as well as others have shown that male and female farmers may differ in terms of when and where they prefer training to be conducted and whether training is male- or female-led (**20-24**). "*

*Also based on the reviewer's comment on ethics, we have added the following to the discussion. Thank you again for pointing out this interesting topic.*

*"Machine supported decision making could help address some of these potential ethical dilemmas that policy makers face when creating gender inclusivity goals by allowing them to first simulate "what if" scenarios before applying policies in the real-world"*

7) My detailed comments are as follows:
Abstract of this study needs to be rewritten with the actual theme of the study, policy recommendations should be provided. I would recommend to expand it with more information about the topic.

*We modified the abstract to better capture the theme of the study and to introduce the ethical dilemma that policy makers face when designing gender inclusivity policies. These are complex issues that would benefit from machine supported decision making. But in the end, the decision making remains with policy makers. Data-driven modelling must not usurp policy makers but instead be a tool for them to simulate "what if" scenarios to make more informed policies. Therefore, we are reluctant to provide specific policy recommendations in the paper.*

*"Women comprise a significant portion of the agricultural workforce in developing countries but are often less likely to attend government sponsored training events. The objective of this study was to assess the feasibility of using machine supported decision making to increase overall training turnout while enhancing gender inclusivity. Using data obtained from 1,067 agricultural extension training events in Bangladesh (130,690 farmers), models were created to assess gender specific training preferences. Using these models, simulations were performed to predict the top (most attended) training events for increasing total attendance (male and female combined) and female attendance, based on gender of the trainer, and when and where training took place. By selecting a mixture of the top training events for total attendance and female attendance, simulations indicate that total and female attendance can be concurrently increased. However, strongly emphasizing female participation can have negative consequences by reducing overall turnout, thus creating an ethical dilemma for policy makers. In addition to balancing the need for increasing overall training turnout with increased female representation, a balance between model performance and machine learning is needed. Model performance can be enhanced by reducing training variety to a few of the top training events. But given that models are early in development, more training variety is recommended to provide a larger solution space to find more optimal solutions that will lead to better future performance. Simulations show that selecting the top 25 training events for total and female attendance can increase female participation by over 82% while at the same time increasing total turnout by 14%. In conclusion, this study supports the use of machine supported decision making when developing gender inclusivity policies in agriculture extension services and lays the foundation for future applications of machine learning in this area. "*

8) Authors are required to clearly explain the study objectives/research questions in the last paragraph of introduction part. What is the contribution of this paper? What are the novel points?

The theoretical framework is weakly described. The research hypothesis are not provided, it should be revised and presented in an articulated way with more updated citations. Further, it is recommended to provide the framework in picture/figure form (using MS. Visio).

*We agree with the reviewer that the study objectives needed to be more clearly stated in order to set the correct expectations for the readers. Significant changes to the manuscript have been made to focus the paper. Also, the research hypothesis is provided in the last paragraph of the introduction.*

*"We hypothesize that machine supported decision making that accounts for gender-based training preferences can concurrently increase total and female participation over that of the current approach that relies only on human intuition. If results support this hypothesis, incorporating machine learning as part of ICTs, and other modes of information dissemination, in large-scale agricultural extension programs is warranted."*

*Also, a section was added to the Materials and Methods section to clearly present the study's central hypothesis.*

*"Evaluated hypothesis.*

*The objective of this study is to provide evidence that machine supported decision making for selecting training events can improve the current approach, based on human intuition. As a benchmark, the average total attendance and average female attendance across all past extension training events will be used. If the machine learning approach significantly exceeds these averages, it provides strong justification for the addition of machine supported decision making in extension services. "*

*To our knowledge, this is the first attempt to apply machine learning to improve participation in agricultural extension training, making it a novel application. A sentence was added to the introduction stating this.*

*"Although there is considerable research into the use of machine learning in agriculture, the applications mainly focus on crop, livestock, water and soil management (**25**); there does not appear to be applications using machine learning to improve agricultural extension participation, including promoting gender inclusivity."*

9) Literature review section is missing, authors are required to add a “Literature review” section.

*We added the following to the introduction.*

*"Although there is considerable research into the use of machine learning in agriculture, the applications mainly focus on crop, livestock, water and soil management (**25**); there does not appear to be applications using machine learning to improve agricultural extension participation, including promoting gender inclusivity."*

10) It is advised to revise the model settings, and parameters presented in this study.

*It is not clear to the authors what is meant by revising the model settings and parameters. We applied a systematic approach to choose the model inputs and converge on parameters used to map model inputs into model outputs. Is the reviewer implying that the machine learning approach was inappropriate to generate the models? We would argue that the machine learning approach is a study strength, which if followed would yield robust modelling results.*

11) The authors need to provide enough literature review to support your study hypothesis, literature in the table form would be more good.

*We added the following to the introduction to support the study hypothesis.*

*"While ICTs increase access to agricultural extension services for production (**17**,* *18**), their deployment must take into account gender-based training preferences. Recent analysis of ICT-delivered agricultural training indicates that differences in male and female farmers exists (**19**). This study as well as others have shown that male and female farmers may differ in terms of when and where they prefer training to be conducted and whether training is male- or female-led (**20-24**). As such, choosing times in the day, days of the week, locations and venues, and gender of the trainer to promote female participation may thwart efforts to increase overall attendance at government sponsored training events (**19**). Therefore, designing effective agricultural training programs that may include multiple objectives, (e.g., increasing general attendance while maintaining a certain proportion of female participation), may not be trivial.*

*More formal mathematical approaches for selecting times, locations and venue types, and gender to conduct training may help improve agricultural training participation and gender inclusivity. Although there is considerable research into the use of machine learning in agriculture, the applications mainly focus on crop, livestock, water and soil management (**25**); there does not appear to be applications using machine learning to improve agricultural extension participation, including promoting gender inclusivity. We hypothesize that* machine supported decision making that accounts *for gender-based training preferences can concurrently increase total and female participation over that of the current approach that relies only on human intuition. If results support this hypothesis, incorporating machine learning as part of ICTs, and other modes of information dissemination, in large-scale agricultural extension programs is warranted."*

*Given that there is no extensive literature in this area, a table may not be necessary.*

11) I have noticed several typo errors throughout the manuscript, further this manuscript is poorly organized, more work need to be done on writing style and organization of the paper.

*We have significantly revised the manuscript to improve readability and reviewed it for grammatical mistakes.*

**Reviewer #2:** 
This paper studies an important subject, related to how we can promote gender inclusivity in agricultural trainings. It is demonstrating a data-driven approach to maximize participation. This could be a valuable contribution for practitioners involved in planning agricultural trainings, and I think the contribution would go beyond trainings on ICTs (which the authors may want to highlight), but I have a few concerns related to the paper.

*Thank you for your positive feedback and providing time to review the manuscript. As the reviewer suggests, we added the following to the introduction to expand the scope of the topic beyond ICTs.*

*"We hypothesize that machine supported decision making that accounts for gender-based training preferences can concurrently increase total and female participation over that of the current approach that relies only on human intuition. If results support this hypothesis, incorporating machine learning as part of ICTs, and other modes of information dissemination, in large-scale agricultural extension programs is warranted."*

12) First, attributes of a training such as the gender of the trainer or the time of the day during which it is organized, could be correlated with unobserved variables such as the workload that prospective participants might face at different times of the day - and thereby agricultural cropping patterns or responsibilities in a given location outside of the house, or the availability of female trainers in a given union or upazila. The variables that are entered in the model are not exogenous, and I am very concerned about omitted variable bias in this case. This warrants more discussion as a limitation in the manuscript, including a discussion on page 13 on how experimentation with some of these variables could give one the exogenous variation that one needs to really say something about the effects of a male versus female trainer, or the timing at which an event is organized. In general, interpreting the presented data and models as information on "training preferences" and to speak of "impacts" really seems like a stretch.

*We agree with the reviewer that marginal information is captured and that a more systematic approach for data collection to reduce omitted variable bias and to improve model performance is recommended, including recording information on unobserved factors, such as those listed by the reviewer. We also agree that experimentally varying these exogenous factors could provide additional insights on training preferences. We have added the following to the discussion to address these points.*

*"... Finally, this observational study likely missed other important factors impacting training turnout. Future data collection should consider investigating potential underlying causes impacting training preferences, such as the workload that prospective participants might face at different times of the day or days of the week, agricultural cropping patterns, and the use of incentives or other recruiting strategies (e.g., female-promoted events)."*

*"	To facilitate machine learning, continuous sampling and processing of data of agricultural training programs are recommended. That is (1) host training events, (2) learn (computationally) from those events, (3) enact recommendations for the next set of training events, and repeat to optimize outcomes. In subsequent rounds of data collection, the project could shift from being purely observational to using optimal experimental design (**33**) to focus data collection to help refine models further and to improve their performance."*

*Acknowledging these limitations, the data collected does provide a useful starting point for modeling and captures important factors that drive male and female attendance. Although, the underlying causes of these gender training differences may not be revealed with the current data collection approach, clear differences between males and females exist. Further, by incorporating the model shrinkage approach, that takes into account generalizability of model results in the test datasets, there is a higher likelihood for these gender training differences to represent real-world differences.*

13) Second, and potentially related, it seems to me that there are crucial factors that might influence participation are excluded. For instance incentives (for instance transport money or whether people are provided with money versus meals) should be a key determinant of participation, and also, I would imagine that the way in which a training is introduced to participants, for instance whether men or women are recruiting the participants, or the communication channels through which the events are being announced, could greatly influence participation. Do the authors have any data on this that could be explored? That would enrich the model and potentially help address some of the concerns about unobserved confounding characteristics in my previous comment. Even without such data, more insights on how participants were recruited / invited to the training sessions seems important context that is lacking at the moment.

*Unfortunately, these variables were not collected as part of the original data collection. But we agree that these and other variables could be collected in the future and the model updated to explore their impact on training turnout.*

*The following was added to the methods describing recruitment.*

*"Training locations and venue types for video presentations were purposefully selected to maximize attendance in order to disseminate information on these topics in advance of the primary maize, rice, and wheat cropping seasons. No incentives were provided to participate (e.g., no meals, no payments). Most events were not pre-organized to recruit participants, but rather involved setting up the video show and attracting a crowd."*

*The following was added to the discussion.*

*" ... Finally, this observational study likely missed other important factors impacting training turnout. Future data collection should consider investigating potential underlying causes impacting training preferences, such as the workload that prospective participants might face at different times of the day or days of the week, agricultural cropping patterns, and the use of incentives or other recruiting strategies (e.g., female-promoted events)."*

14) Third, whilst I understand that division is an important correlate of participation, it leads to somewhat weird conclusions. For instance, Rajshahi was selected frequently in the top female training list. Does this mean that we should organize more training sessions in Rajshahi, and ask women from other divisions to come there? Or simply ignore women in other divisions? I'm sure this is not what the authors have in mind. It might be good to predict attendance irrespective of divisions, so as a first step, look at what types of events would increase turnout and female participation across all divisions.

*This is an excellent point, which warrants some discussion in the paper. As the reviewer suggests, one could ignore the division factor and create a generic country model. But this would likely reduce the accuracy in model predictions, which would then reduce model performance to increase total and female training participation. Alternatively, optimization of training selection could be done with constraints, such as the requirement to select equal number of training events from each division. This will also likely impact model performance and needs to be taken into account when creating gender inclusivity policies. We have added the following to the Discussion section to address this point.*

*"The objective of this study was to determine if machine supported decision making could improve training attendance and gender inclusivity, which would then justify its use in designing large-scale agricultural training programs. The results suggest that, if gender inclusivity goals are sensibly designed, machine supported decision making can concurrently increase total attendance and gender inclusivity. The results, however, also show that overly emphasizing female participation can have negative consequences for total training turnout. For instance, selecting all the training events from the top female list would increase female attendance and the proportion of female attendance to ~ 0.5 but could decrease total attendance below that of the benchmark average of 122 attendees per event (Figure 1). Moreover, many of the top training events for females are predicted to take place in Rajshahi, and therefore, focusing on this one division may be beneficial for overall female attendance, but it would limit gains in female participation to one region, which may not fulfil the goals of the extension program. Machine supported decision making could help address some of these potential ethical dilemmas that policy makers face when creating gender inclusivity goals by allowing them to first simulate "what if" scenarios before applying policies in the real-world."*

15) Fourth, the discussion on "reducing training variability from lists of 100 to 50 to 10" is unclear. I think this is related to the comment that one needs to balance machine learning with performance, and that overemphasizing early model performance may restrict learning, but this point could be clarified more.

*Based on the reviewer's comments, we have added the following sentence to the Materials and Methods section to improve clarity.*

*"	An algorithm was created to simulate the effects of weighting the importance of female participation over total attendance. This was done by selecting the top X% from the female list and replacing the lower X% from the total list, where X% represents a range (0%-100% with 10% increments). For example, the top 50%, reflecting a balance between increasing total attendance and increasing female participation, would take the top 50 training events from the total list and the top 50 training events from the female list to create a new list of 100. The new list would then be used to estimate the average total attendance, female attendance, and proportion of female attendance. This was done to qualitatively capture the effects of weighting female attendance over total attendance to help develop sensible gender inclusivity goals.*

*Reduced training variety, representing smaller lists of the top training events, is expected to improve model performance and lead to greater total attendance, female attendance, and proportion of female attendance; however reducing training variety may also limit future model performance. Because the models are still in their infancy, future iterations with more observations are expected to improve machine learning and subsequent model performance. At this point, selecting a few of the top attended events may restrict machine learning and lead to suboptimal future results. Greater training variety provides a larger solution space and increases the odds of finding the optimal solution. To explore the trade-off between model performance and machine learning, simulations were performed with lists of the top 100, top 50, and top 10 training events for total and female attendance. This was done to qualitatively capture the effects of training variety on total attendance, female attendance, and proportion of female attendance. "*

16) Finally, given that the number of variables on page 18 is not that large, and most interaction terms appear being dropped using the variable reduction methods, I'm wondering what the added value is of using this data-driven approach as opposed to just estimating a Poisson count model in STATA or R, and indeed, it would be good to see those regressions for the reader to see how the different variables from page 18 are related to participation.

*Documenting the relationship between different variables on training attendance using regression analysis was completed in a prior publication. We expand on this earlier work by performing simulations to explore the relationship of different gender inclusivity policies and to study the trade-off between model performance and machine learning. This publication was included in the initial submission of the current manuscript. We now refer to this published work in the introduction and use it to justify the machine learning approach.*

*"While ICTs increase access to agricultural extension services for production (**17**,* *18**), their deployment must take into account gender-based training preferences. Recent analysis of ICT-delivered agricultural training indicates that differences in male and female farmers exists (**19**). This study as well as others have shown that male and female farmers may differ in terms of when and where they prefer training to be conducted and whether training is male- or female-led (**20-24**). As such, choosing times in the day, days of the week, locations and venues, and gender of the trainer to promote female participation may thwart efforts to increase overall attendance at government sponsored training events (**19**). Therefore, designing effective agricultural training programs that may include multiple objectives, (e.g., increasing general attendance while maintaining a certain proportion of female participation), may not be trivial. "*

17) A minor comment: The sessions that were dropped were not different in size, at least not significantly so, from the sessions that were not dropped. This might be worth emphasizing in the manuscript.

*Thank you for raising this point. We have added a sentence to the manuscript to emphasize that the sessions that were dropped were not significantly different in size.*

*"* *... Based on a t-test, there was no evidence of difference in the average number of male or female attendance between the included and not included training sessions. "*
