# Supplementary material for: Machine-supported decision-making to improve agricultural training participation and gender inclusivity
Source: PLoS One. 2023 May 5;18(5):e0281428. doi: 10.1371/journal.pone.0281428 (PMC10162538; doi:10.1371/journal.pone.0281428)
Supplement: Attachment — Submitted filename: Reviewer Response pr9_tk1_gc1.docx [file pone.0281428.s003.docx]

**PlosOne**

**Point-by-Point Response to Reviewers**

PONE-D-22-16835R1

Machine Supported Decision Making to Improve Agricultural Training Participation and Gender Inclusivity

*We thank the editor and reviewers for their constructive feedback. Changes to the paper were made based on these comments (see the point-by-point response below). Our comments are in italics. Parts taken from the paper are in quotation marks (“ ”), and new sections added are underlined.*

**Responses to Editor's Comments:**

#1. Please take particular care to the comments raised by Reviewer 2(R2) .

*See response #8, #9, and #10.*

#2. Please explicitly provide clearer motivations on the use of a machine learning approach over simpler models.

*See response #8 and Supplemental Material Part B.*

#3. Explain better the used training split for the data and the other choices employed in this step.

*Typically with small datasets, such as the ~ 1,000 training events collected in the current study, a higher portion of the data (e.g., 0.8) is used for the training dataset to ensure that the proper parameters are selected for the model and their coefficients properly estimated. We completed additional analysis to determine if different training/testing splits affect the accuracy of the model. We have shown that the accuracy is consistent amongst different training/testing splits. This additional analysis is included in the Supplemental Material Part B.*

*In addition to the above, please see response #8 for details regarding the motivation for using the machine learning approach versus the more simple regression model used in the previous publication, and please see the Supplemental Material Part A for a more thorough comparison of the 3 model shrinkage approaches and Supplemental Material Part B for a comparison of 2 different regression models. Results show that model accuracy is consistent across shrinkage approaches and regression models.*

#4. Provide more proof about the generalization capability of the used methodology, providing a clearer validity of the overall approach.

*Based on the reviewer's concern, she/he indicated that it may not be feasible to use the proposed modeling approach given that it would lead to most of the training occurring in one division, which may not be desirable.*

*Briefly, we demonstrate that the models that incorporate Bangladesh division can be used for regionally balanced training selection (i.e., balancing training across all divisions). Follow-up simulations show that balancing training across all divisions does not significantly reduce overall attendance or female participation. These findings support that the approach can be applied to ensure equal access to training, and supports its generalizability in a real world context.*

*See response #9 and Supplemental Material Part C for more details.*

**Journal Requirements:**

#5. Please take carefully into account all the comments for improving the manuscript to meet PLOS ONE standards before resubmitting it to the journal.

*The manuscript complies with PLOS ONE standards.*

**Responses to Reviewers Comments**

**Reviewer #1:**

#6. This manuscript can be accepted in current form, as the authors have well addressed all the concerns.

*Thank you for your time reviewing. Your comments were very helpful in improving the manuscript.*

**Reviewer #2:**

#7. I thank the authors for considering my comments, and for their thoughtful response. I believe that the paper has improved in that it is more nuanced, and is improved in its readability. At the same time, it seems like some of my comments are not fully addressed. I suggested additional analyses that were not done, without good reason for not doing so.

*Thank you for your effort in reviewing and constructive feedback, which has helped to improve the manuscript. We apologize for not fully addressing your previous comments.*

*To better address your comments, we have conducted additional analyses. These analyses include the following:*

1. *More thorough comparison between model shrinkage approaches and regression models.*
2. *Evaluation of the improvements in terms of accuracy with the new approach that allows a machine to select parameters for the model versus the past simple regression model in which humans selected parameters.*
3. *Assessment of the impact of balancing training events across divisions on overall turnout and female participation.*

*The analyses is included in the Supplemental Material. Also, see response to #8 and #9 for more details regarding the additional analyses.*

#8. I am still concerned about the added value of the machine learning approach over and beyond a simple regression model. The regression was already estimated for another paper, but that is not a reason for not comparing the results from the machine learning model with the predictions by that regression model, and pointing out explicitly what is changed by the ML model.

*In the previous publication, humans selected the parameters to be incorporated into the model, focusing only on main effects and their associations with total attendance and female participation. In the new manuscript, we broadened the scope of the model to include main effects and their interactions. We then allowed the machine using model shrinkage to select a smaller subset of parameters (i.e., main effects and interactions) that were important to predict total attendance and female participation. This was done to improve the generalizability of the model by avoiding over-fitting of data.*

*As suggested by the reviewer, we have completed additional analysis to compare the regression model in the previous paper with the regression model generated from the machine learning approach. Results show that the machine learning approach using model shrinkage generated less error than the previous regression model for predicting male attendance and produced similar error for predicting female attendance. We suspect that broadening the model to include interactions was favorable for the male model but not for the female model, which did not select any interactions. In the male model, there was an interaction between division and time of day, suggesting that the best time of day for training to increase male turnout varied amongst divisions. For females, the best time of day for training appears to be more consistent across divisions. Error comparisons between the new and past models are included as Supplemental Material Part B.*

*We would argue that using the machine learning approach is useful for revealing underlying patterns in the data that may be difficult for humans to observe. This is particularly true for predicting the impact of gender inclusivity policies. The main contribution of the paper is the use of model simulations to explore sensible gender inclusivity policies. We show how machine supported decision making can be used by policy makers to improve not only female attendance but also overall attendance at extension training events, something that may be difficult to achieve using human intuition. This is the key contribution of the paper, which was not explored in the previous paper.*

#9. Further, I am still concerned about the context-specificity, and overpredicting to a specific context, with awkward conclusions such as "policymakers can maximize turnout of female farmers by organizing trainings in such and such area". Policymakers would want a more generalizable model, which they can apply to a country as a whole, even if at the cost of lower accuracy. Showing how much accuracy is lost by having a slightly more generalizable model would be extremely valuable in demonstrating how to best employ this ML approach, and do a bit more to address my concern than just including a sentence or two discussing the limitation - I am worried that those few sentences are too easily missed.

*Thank you for this comment and we apologize for not addressing this issue in a satisfactory way. We reviewed your previous comments, which stated:*

...whilst I understand that division is an important correlate of participation, it leads to somewhat weird conclusions. For instance, Rajshahi was selected frequently in the top female training list. Does this mean that we should organize more training sessions in Rajshahi, and ask women from other divisions to come there? Or simply ignore women in other divisions? I'm sure this is not what the authors have in mind. It might be good to predict attendance irrespective of divisions, so as a first step, look at what types of events would increase turnout and female participation across all divisions.

*We agree with the reviewer that not enough was done to address this important point.*

*Division is a strong predictor influencing both total turnout and female participation. In addition, the male model includes an interaction between Bangladesh division and time of day, which suggests that optimal times for training in one division may not be favorable for other divisions. Further, discussions with our Bangladesh colleagues suggests that agricultural extension planners tend to focus their activities in specific geographic locations rather than nationally, which would also favor keeping divisions in the model to make it more regionally relevant.*

*Our opinion is that it is better to develop a more accurate model and add geographical constraints during training selection. For instance, policy makers could select the top training events regardless of division or they could impose a constraint such as selecting equal numbers of training events from each division to balance training across the country. We have completed these simulations and have shown that balancing training across all divisions does not significantly impact total training turnout and female participation, which supports the adoption of the policy. This additional analysis is added in the Supplemental Material Part C.*

*The following has been added to the discussion:*

*"... Moreover, many of the top training events for females are predicted to take place in Rajshahi, and therefore, focusing on this one division may be beneficial for overall female attendance, but it would limit gains in female participation to one region, which may not fulfil the goals of the extension program. Follow-up simulations show that balancing training across all divisions does not dramatically reduce overall attendance or female participation, and still exceeds total and female averages based on human intuition (see Supplemental Material). As shown in this study, machine supported decision making could provide important insights by allowing policy makers to first simulate "what if" scenarios before applying policies in the real-world."*

#10. Finally, the manuscript still speaks of training "preferences" when in fact there is too much omitted variable bias and too little control over the invitation process to be able to interpret turnout as "preferences". What is called "preferences" could simply be "presence" when the events are being marketed and a crowd is attracted; or "permission to go check out what's happening", or "time availability", correlated with the determinants going into the ML model, and I really think the word "preferences" should not be used here.

*We apologize for not understanding your concern that was raised in the prior review. We agree with you that the use of "preferences" is not appropriate. Instead, we have changed the term to "patterns", which suggests there are patterns to the training data, but not necessarily reflecting the preferences of the attendees. Thank you for raising this issue again; it was important to address correctly.*

*Abstract*

*"Using data obtained from 1,067 agricultural extension training events in Bangladesh (130,690 farmers), models were created to assess gender-based training patterns (e.g., preferences and availability for training)."*

*Introductions*

*"While ICTs increase access to agricultural extension services for production (**17**,* *18**), their deployment likely would be enhanced by taking into account gender-based training patterns (e.g., preferences and availability for training)."*

*"We hypothesize that machine supported decision making that accounts for gender-based training patterns can concurrently increase total and female participation over that of the current approach that relies only on human intuition."*

*Materials and methods*

*"Next, using the most influential model inputs that were derived for the male and female models respectively, generalized linear models were developed to capture gender-based training patterns."*

*Discussion*

*"Future data collection should consider investigating potential underlying causes impacting gender-based training patterns, such as the workload that prospective participants might face at different times of the day or days of the week, agricultural cropping patterns, and the use of incentives or other recruiting strategies (e.g., female-promoted events)."*
